# Cell Lineage Affiliation During Hematopoiesis

**DOI:** 10.3390/ijms26073346

**Published:** 2025-04-03

**Authors:** Geoffrey Brown

**Affiliations:** Department of Biomedical Sciences, School of Infection, Inflammation, and Immunology, College of Medicine and Health, University of Birmingham, Edgbaston, Birmingham B15 2TT, UK; g.brown@bham.ac.uk; Tel.: +44-(0)121-414-4082

**Keywords:** hematopoiesis, hematopoietic stem cells, lineage affiliation, differentiation, self-renewal

## Abstract

By the mid-1960s, hematopoietic stem cells (HSCs) were well described. They generate perhaps the most complex array of functionally mature cells in an adult organism. HSCs and their descendants have been studied extensively, and findings have provided principles that have been applied to the development of many cell systems. However, there are uncertainties about the process of HSC development. They center around when and how HSCs become affiliated with a single-cell lineage. A longstanding view is that this occurs late in development and stepwise via a series of committed oligopotent progenitor cells, which eventually give rise to unipotent progenitors. A very different view is that lineage affiliation can occur as early as within HSCs, and the development of these cells to a mature end cell is then a continuous process. A key consideration is the extent to which lineage-affiliated HSCs self-renew to make a major contribution to hematopoiesis. This review examines the above aspects in relation to our understanding of hematopoiesis.

## 1. Introduction

The stem cell concept dates to the 19th century and HSCs and their descendants have been extensively studied due to their ease of accessibility. Hematopoietic stem cells (HSCs), which are rare cells in the bone marrow, ensure that blood and immune cells are produced each day throughout life to replenish worn-out or short-lived mature cells. For example, around 10^11^ granulocytes are produced each day in humans [1]. The ‘feed’ from HSCs into hematopoiesis is low due to their scarcity, which is coupled with a low level of amplification (see below). To meet the large and continuous demand for mature cells, HSCs self-renew giving rise to identical daughter cells that have not differentiated so that there is a supply of HSCs for the lifespan of an organism.

**Figure 1:**
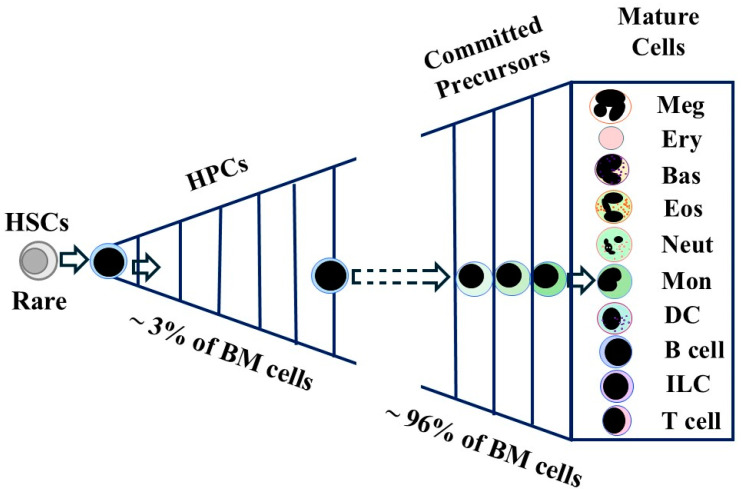
Compartmentalization of hematopoiesis. Self-renewing hematopoietic stem cells (HSCs) give rise to the second tier of cells, which amplifies the number of cells produced by an HSC. This tier includes the hematopoietic progenitor cells (HPCs) and the recognizable precursor cells to each cell type. HPCs divide a substantial number of times to give rise ultimately to the unipotent HPCs. Precursor cells divide to a lesser extent as they mature to give rise to the third tier of functional end cells. Expansion is shown by the triangle with the lines providing an indication of the number of cell cycles. Meg, megakaryocytes; Ery, erythrocytes; Bas, basophils; Eos, eosinophils; Neut, neutrophils; Mon, monocytes; DC, dendritic cells; ILC, innate lymphoid cells.

For many years, hematopoiesis has been seen as a three-tier system with each compartment comprising cells with different attributes, namely self-renewal, the capacity to expand cell numbers from the use of a single HSC, and end-cell functionality (Figure 1). Self-renewing HSCs are the first tier. Offspring that are selected for differentiation give rise to the second tier of cells, which serves to amplify the production of mature cells by means of two cell populations. Hematopoietic progenitor cells (HPCs) divide a considerable number of times (perhaps 20 to 30 cycles); as few as twenty cycles would result in an amplification of 10^6^ times. As these cells divide, they are not thought to give rise to identical offspring and instead age stepwise, via a series of progenitor cells with differing lineage potentials, to eventually give rise to cells that are committed to a single lineage, namely unipotent HPCs. In keeping HPCs, which includes cells that are multipotent progenitors (MPPs), do not engraft a mouse to sustain hematopoiesis long-term. Uni-potent HPCs give rise to the morphologically recognizable precursors, for example, myeloblasts, which are also committed to generating their blood cell type. Myeloblasts divide three to five times to yield the non-dividing metamyelocytes that give rise to mature neutrophils. The third tier consists of functionally mature cells, which do not typically divide, other than lymphocytes.

From HSCs to functional end cells is a steady progression (Figure 1) with controversies centering around whether cells undergo stepwise commitment or development is continuous and at what stage(s) there is self-maintenance of cells. In canonical models of hematopoiesis, HSCs are a homogeneous population of multipotent cells that self-renew. HPCs are a mixture of multipotent progenitor cells (MPPs), cells with differing lineage potentials, and uni-potent cells. It has become increasingly clear that mouse and human HSCs are a mixture of cells whereby lineage affiliation can occur much earlier than thought. Lineage primed/affiliated HSCs have been identified from gene expression studies. HSCs that are biased towards stable and long-term reconstituting of some blood cell types, and just one cell type, have been identified from transplantation studies. These findings have challenged whether (1) self-renewal is an attribute that is unique to HSCs, and (2) lineage affiliation is entirely the domain of MPPs and their offspring. If HSCs can determine their lineage fate directly, there is no longer the need to describe cells as HSCs versus HPCs. This review examines the evidence to support or otherwise different views on hematopoiesis.

## 2. HSCs Are Rare and Quiescent Cells That Self-Renew

A precise figure for the frequency of HSCs in human bone marrow is difficult because marrow aspiration is inconsistent. From surface marker analyses, HSCs are found within the CD34^+^ population of cells, which is heterogeneous, and ~0.7% of the total marrow cells in young adults [2]. The frequency of HSCs within CD34^+^ cells is ~5% (range 2–8%) [3]. The use of the above percentages gives rise to a frequency of ~0.03% for HSCs in bone marrow. A value of ~11,000 cells has been calculated for the reserve of human HSCs in adolescent marrow [4]. The frequency of mouse HSCs in bone marrow is 1 in 10^4^ as determined by transplantation and long-term repopulation studies [5]. A frequency of 2–5 HSCs per 10^5^ total bone marrow cells was obtained for adult mouse bone marrow from a study that looked at HSCs that were most productive and that had a durable self-renewal potential [6,7]. The frequency of mouse HSCs is higher at ~0.01% of nucleated cells based on the expression of stem cell-affiliated surface markers, with ~5000 HSCs obtained from a mouse [8]. Both human and mouse HSCs are largely quiescent. The percentage of human HSCs that are cycling (non-G0) is around 10 (range 5–15) [3] and they replicate on average around once every 40 weeks (range 25 to 50). Mouse HSCs replicate on average once every 2.5 weeks [4].

HSCs are often mistaken as immortal cells. Transplantation studies showed that HSCs generate erythrocytes and lymphocytes throughout the lifespan of a mouse leading to the extensive lifespan of HSCs [9]. Blood cell reconstitution has been repeated for three to four mouse lifespans in serial transplantation studies [10]. To account for this self-renewal, a longstanding view is that HSCs reside in a specialized perivascular niche in the bone marrow. The niche provides signals that are crucial to self-renewal and is created partly by mesenchymal cells and endothelial cells. However, it is much more complex— there is no singular niche cell, and other influences include, for example, non-myelinating Schwann cells, osteoblasts, macrophages, and the blood reviewed in [11]. Even so, whether HSCs are truly immortal is a futile argument because self-renewal has not been demonstrated for tens of thousands of divisions and each HSC probably divides just some two to three hundred times in four mouse lifespans (divides ~ every 2.5 weeks). Accumulated genetic defects from endless cell divisions, and/or as HSCs age, may decrease the capacity for repair and, in turn, the fidelity of self-renewal [12].

## 3. Are HSCs Homogenous or a Mixture of Cells?

### 3.1. The HSC Compartment Is a Homogeneous Population of Multipotent Cells

In a ground-breaking series of experiments in the 1960s, Till and McCulloch used sublethal irradiation to induce clonal markers in bone marrow cells. When these cells were transplanted into a mouse, they gave rise to visible clones of marked cells in the spleen (spleen colony forming units, CFU-S) that contained a mixture of granulocytes, macrophages, erythrocytes, and megakaryocytes. Some CFU-S cells had replicated themselves because transferring CFU-S cells to an irradiated host produced more CFU-S, with the above mixture of cells, and some spleens contained cells with the potential to make lymphocytes [13,14,15]. Till and McCulloch proposed that these multipotent and self-renewing cells are HSCs. Subsequently, mouse HSCs were purified as cells that lacked the lineage-specific markers CD4 (T cells), CD8 (T cells), B22 (B cells), Gr-1 (granulocytes), Mac-1 (macrophages) and TER119 (erythrocytes), abbreviated as Lin^−^, and cells that expressed Sca-1^+^ (a stem cell marker) and c-kit^+^ (a stem cell growth factor receptor). The most primitive HSCs were found within Lin^−^, Sca-1^+^, c-kit^+^ (LSK) cells that were low to negative for expression of CD34 (a stem/progenitor cell marker). In 1996, the injection of a single LSK, CD34^low/−^ cell into a mouse led to the long-term reconstitution of myeloid and lymphoid cells in 21% of recipients [16]. A more efficient method for the transplantation of LSK, CD34^−^ mouse HSCs was provided by culturing sorted individual cells in mouse c-kit ligand and either mouse interleukin-11 or human recombinant G-CSF. Transplantation of clones of <15 cells showed high-level multilineage reconstitution [17]. Clearly, multipotent HSCs that self-renew reside at the top of the hematopoietic hierarchy. 

A longstanding view is that self-renewal is unique to HSCs. From transplantation studies, HSCs that repopulate all cell lineages have been classified as long-term HSCs (LT-HSCs), intermediate-term HSCs (IT-LSCs), and short-term HSCs (ST-HSCs) [18]. These cells are phenotypically HSCs, and the level and duration of reconstitution varies for the three sub-types. LT-HSCs sustain hematopoiesis for the lifespan of a mouse. ST-HSCs generate all blood cells but for only several weeks, and in this case do not repopulate with absolute efficiency [19]. When ST-HSCs are secondary transplanted they generally do not show any constitution, whereas reconstitution levels do not change for secondary transplantation of LT-HSCs. As mice age, the number of HSCs increases but their ability to repopulate a mouse decreases [20]. The capacity for self-renewal is, therefore, a variable trait within HSCs, and Hoxb5 plays a role in imparting this functional heterogeneity. For Hoxb5-negative HSCs, exogenous *Hoxb5* expression conferred protection against the loss of self-renewal capacity and partially altered the fate of ST-HSCs to that of LT-HSCs [21].

Are HSCs the sole hematopoietic cells with the capacity to self-renew? Erythroid progenitors from the bone marrow of adult mice expand 10^2^-to 10^5^-fold when grown in the presence of erythropoietin, in keeping with their role to expand cell numbers in vivo. Culture of cells obtained from E9.5 mouse yolk sac in serum-free conditions generated immature erythroblasts, which expanded 10^10^- to 10^30^-fold, and at best 10^64^-fold. The cells that were extensively renewed were definitive erythroid (Ter119^low^), c-kit^high^, and eventually matured into enucleated erythrocytes [22]. The following studies provided clear evidence that loss of self-renewal is not fundamental to the affiliation of HSCs to a cell lineage(s). Mouse HSCs are more stringently defined than human cells and often as LSK CD150^+^ CD48^−^ CD34^−^ cells. LSK, CD150^+^, CD48^−^, CD34^−^ sorted cells were cultured as single cells in the presence of stem cell factor and thrombopoietin, which is the major cytokine to megakaryocyte development. Daughter pairs were micromanipulated, and each cell was transplanted into lethally irradiated mice to examine their reconstitution nature. For some pairs, both daughter cells reconstituted hematopoiesis whereby the single cell had undergone a symmetric cell division to give rise to HSCs (combinations of a LT-HSC, IT-HSC and ST-HSC). Distinct patterns were observed for some pairs whereby the single cell had undergone an asymmetric cell division to give rise to either an HSC (LT-HSC or ST-HSC) and a megakaryocyte-restricted repopulating cell or an HSC (ST-HSC) and a common myeloid-restricted (megakaryocyte, erythroid, and myeloid) repopulating cell. HSCs had generated lineage-restricted cells directly that were able to self-renew to a considerable extent [18]. Altogether, the above findings support the view that self-maintenance is not restricted to HSCs. This, in turn, blurs any clear distinction between cells that we compartmentalize as HSCs versus HPCs.

### 3.2. HSCs Are a Complex Mixture of Cells

Early studies used single-cell RT-PCR to examine HSC heterogeneity regarding the expression of cytokine receptors by mouse multipotent FDCP-mix A4 cells. Self-renewal of these cells was maintained by culturing in interleukin 3. There was variable low-level expression of mRNAs for the receptors for the lineage-affiliated cytokines erythropoietin, granulocyte-colony stimulating factor, granulocyte/macrophage colony-stimulating factor, and macrophage colony-stimulating factor (M-CSF). There was a high degree of cell heterogeneity regarding co-expression. Therefore, the expression of lineage-affiliated genes occurs in the self-renewal state with the investigators concluding that promiscuous lineage priming occurs prior to HSC lineage commitment [23]. From single-cell RT-PCR studies of mouse HSCs, ~12% of LT-HSC (LSK CD150^+^ CD48^−^, CD34^−^) and ~20% of ST-HSC (LSK CD150^+^ CD48^−^ CD34^+^) expressed mRNA for the erythropoietin receptor, and ~19% of LT-HSC and ~24% of ST-HSC expressed the receptor for macrophage colony-stimulating factor (M-CSFR) at their surface. The fms-like tyrosine kinase receptor (Flt3) was expressed at the cell surface by ~5% of LT-HSC and ~8% of ST-HSC. Co-expression of the mRNAs encoding Flt3 and the erythropoietin receptor was rarely seen whereas co-expression of Flt3 and M-CSFR was observed by 1 to 3% of the M-CSFR positive cells. LT-HSC and ST-HSCs are, therefore, a heterogeneous population of cells [24].

A low level/priming of gene expression may reflect either a clear inclination to adopt a developmental pathway or be insignificant if such relates to noise within gene expression. Regarding inclination and physiological importance, it is important to note that growth factors for some of the above receptors can direct the fate of HSCs/HPCs. Erythropoietin has been shown to guide HSCs toward an erythroid fate [25], and M-CSF has been shown to instruct granulocyte/macrophage HPCs to adopt a macrophage fate [26]. Additionally, the expression of the receptors for M-CSF and granulocyte colony-stimulating factor is autoregulated, which might serve to enhance low-level expression [27].

Evidence for lineage biases within HSCs has also been provided from transplantation studies. Adolphson and colleagues examined heterogeneity within LSK HSCs by using Flt3 as a surface marker. LSK KSCs that expressed a Flt3 at a high level had a high proliferative potential and sustained granulocyte, monocyte, and B and T cell production and LSK HSCs that did not express Flt3 failed to produce significant erythroid and megakaryocytic progeny. Downregulation of genes that encoded regulators of erythroid and megakaryocyte development accompanied distinct lineage restriction. The Flt3^high^ cells were termed lymphoid-myeloid HSCs, and the investigators proposed a revised road map for hematopoiesis [28]. Muller-Sieburg and colleagues obtained clones of HSCs from mouse bone marrow by limiting dilution on a stromal cell (S17cells) and examined their lineage potentials by transplantation into lethally irradiated mice. From this approach, they described HSCs with an extensive self-renewal capacity that were myeloid biased. These cells showed an impaired responsiveness to interleukin-7, a cytokine that is critical for B cell, T cell, and innate lymphoid cell generation [29]. In aged mice, there is a skewing of HSCs towards myeloid development [30]. This may be attributable to a genetic change, but other investigators have argued that a change to the cells’ environment may contribute [31]. Montecino-Rodriguez and colleagues described a lymphoid-biased mouse HSC (LSK CD150^low^ CD135^−^) that efficiently generates lymphoid progeny and is maintained with age [32]. The expression of CD201 has been associated with lymphoid-biased HSCs [33]. Sanjuan and colleagues identified a megakaryocyte-primed subset of mouse HSCs (LSK CD150^+^ CD48^−^ CD34^−^) by virtue of expression of the platelet-associated von Willebrand factor, which is involved in platelet aggregation. The primed cells showed a propensity for short- and long-term platelet reconstitution, and thrombopoietin, a cytokine that primarily regulates megakaryocyte development, was required for their maintenance. The investigators proposed that megakaryocyte-biased HSCs reside at the top of hematopoiesis [34].

Myeloid-biased HSCs have been described for human HSCs, and as for mouse HSCs, there is HSC skewing towards myeloid development and a decline in B cell lymphopoiesis in old age [3]. As seen for mouse platelet-biased HSCs, a subset of human adult bone marrow HSCs (CD34^+^CD38^−/dim^ cells) was identified that expresses the receptor for thrombopoietin [35]. To investigate heterogeneity within primitive CD34^+^ HSCs from human cord blood, cells were CD49f^+^ index-sorted, lentivirus barcoded, and pools of transduced cells were injected into irradiated mice to examine their lineage potentials. Few clones (8% at 30–38 weeks) yielded cells of many lineages, and, strikingly, 30% produced B cells ± CD34^+^ cells [36]. Therefore, both human and mouse HSCs can be organized into subsets, and we are yet to learn about more subsets.

## 4. What Are the Lineage Options That Are Available to HSCs?

### 4.1. HSCs Choose to Develop Towards Either Myeloid or Lymphoid Cells

In 1997, Kondo and colleagues described a clonogenic cell that was present in adult mouse bone marrow (Lin^−^, Sca-1^lo^, c-Kit^lo^, IL-7R^+^, Thy-1^−^) that had a rapid reconstitution capacity in vivo that was restricted to B cells, T cells, and natural killer cells. The cell lacked myeloid differentiation potential in vitro, and is, therefore, a common lymphoid progenitor (CLP) [37]. A mouse cell that gave rise to either megakaryocyte/erythrocyte or granulocyte/macrophage progenitors, the common myeloid progenitor (CMP), was described in 2000 [38]. A feature of canonical models is the presence of a CLP and a CMP whereby HSCs/MPPs make an immediate choice to differentiate towards either lymphoid or myeloid cells.

### 4.2. A Continuum View of the Options That Are Available to HSCs

In 2009, a new model for hematopoiesis provided a very different view regarding HSC developmental options [39]. All end options were shown to be available as a continuum of fate choice. The existence of lymphoid-myeloid HSCs contradicts a strict lymphoid/myeloid dichotomy. Moreover, macrophages and B cells are closely related functionally as they are both antigen-processing cells. Comparison of the phosphoproteins that are expressed by human precursor macrophage and B cell lines revealed similar patterns [40]. Other workers derived a macrophage cell line from a pro-B lymphoma line (ABLS8.1) [41] and observed macrophage lineage switching of mouse early pre-B lymphoid cells [42]. Lymphoproliferative disorders in interleukin-7 transgenic mice were an expansion of immature B cells that also have macrophage potential [43]. In 1992, a bipotent precursor of B cells and macrophages was identified in mouse fetal liver [44] and later in adult bone marrow [45].

A further proposal was that HSCs can affiliate directly to a cell lineage. This is supported by mouse and human HSCs that selectively express the receptors for cytokines that are associated with the development of a cell lineage, including for erythropoietin, M-CSF, and thrombopoietin (see above). Knapp and colleagues concluded that multiple directional paths are available to primitive human cord blood cells from analyzing the molecular features of single cells and the presence of unipotent B cell clones (see above). Mouse bone marrow cells were sorted as LSK cells, and single-cell RNA-seq was used to examine their transcriptional landscape. A detailed map for the clustering of single-cell profiles revealed 19 subpopulations that were transcriptionally homogeneous. There was an absence of progenitors with a mixed lineage and the investigators concluded that there was transcriptional priming towards seven different fates, namely neutrophil, basophil, eosinophil, monocyte, dendritic cell, erythroid, and megakaryocyte [46]. Whilst the sorted cells were myeloid HPCs, as described, the findings concur with the direct availability of multiple fates to a developing cell.

However, HSCs have been described as able to self-maintain and are lymphoid/myeloid or lymphoid or myeloid biased. Perhaps this leans towards having to just extend self-maintenance to oligopotent HPCs. In other words, perhaps cells that are at whatever stage of lineage restriction can self-maintain. Recent findings from tracking of HSC differentiation over time, the use of single-cell RNA sequencing, and computational analysis have revealed a complex dynamic to the nature of HSCs and HPCs. These studies showed that self-renewal varies across the HSC/HPC landscape and extends to lineage-specific patterns of self-renewal. Regarding differentiation trajectories, cell fate and subsequent differentiation existed crucially in specific experimental conditions. For example, cells that are described as CMPs rarely show a combined megakaryocyte, erythrocyte, granulocyte, and monocyte output, are primed towards a specific lineage and rarely behave as multipotent cells. CMPs move under strong differentiation conditions towards a particular fate rather than exploring other fate options [47] and rarely showed the above-combined output in the early studies that described CMPs [38]. A final consideration that is often overlooked regarding blood cell production is the extent to which cell pools that are embryonic born contribute to hematopoiesis during early and adult life. Multipotent embryonic hematopoietic progenitors that arise soon after endothelial-to-hematopoietic transition predominantly drive hematopoiesis in the young adult. Their contribution to blood cell production decreases over time but they have a lifelong contribution as a predominant source of lymphocytes [48]. It has also been shown that pools of HSCs and HPCs that are established during embryogenesis self-renew in parallel over life and contribute to blood cell production [49].

As follows, there is a need to look at findings for HSCs as they develop toward an end cell type. Is there a canonical hematopoietic cell lineage tree with a discrete set of irreversible differentiation states that are defined by a series of progenitors with differing lineage potentials? Alternatively, do HSCs start their development from a broad landscape of a continuum of options, are the trajectories broad, and do the controls on lineage development drive choices between adjacent cell fates?

## 5. HSC/HPC Developmental Progression

### 5.1. A Canonical Cell Lineage Tree for Developmental Progression

**Figure 2:**
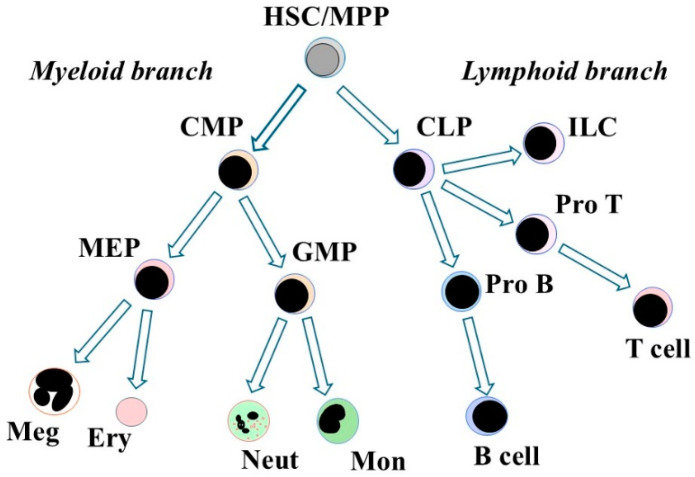
Stepwise restriction of lineage options. Hematopoietic stem cells (HSC) that are undergoing differentiation follow a preferred route to stepwise restrict their lineage options. The choice that an HSC/MPP makes is to develop towards either myeloid or lymphoid cells. Subsequently, hematopoietic progenitors that have different cohorts of options define the model. MPP, multipotent progenitor cell; CMP, common myeloid progenitor cell, CLP, common lymphoid progenitor cell; MEP, megakaryocyte/erythroid progenitor cell; GMP, granulocyte/monocyte progenitor cells, Meg, megakaryocytes; Ery, erythrocytes; Neut, neutrophils, Mon, monocytes; ILC, innate lymphoid cells.

Figure 2 shows a simplified hematopoietic cell lineage tree to illustrate the principle of stepwise restriction of lineage options via a binary decision-making process. Canonical models depict a tree topology [50], and there is a plethora of models regarding the routes that are followed by developing MPPs [39]. In all branching models, HPCs follow a preferred route to stepwise restrict their development towards a particular fate. The routes were mapped largely from the types of HPCs that were observed when bone marrow cells were dispersed in a semi-solid medium whereby individual colony-forming unit (CFU) cells were observed to give rise to colonies that contained various types of cells [51]. Like CMPs, some cells gave rise to colonies containing granulocytes, erythrocytes, monocytes, and megakaryocytes and were termed colony-forming unit (CFU)-GEMM. Other cells gave rise to colonies that contained just two types of cells, for example, granulocytes and macrophages, and were termed CFU-GM. Stepwise commitment that was irreversible was concluded by placing the types of CFUs observed in an order so that they progressively reduced their fate options.

For some canonical models, topologies show various pathways to an end cell type [39]. Early evidence to support the use of different trajectories by HPCs was provided by examining the transcription profiles of dendritic cells that had been derived in vitro from cells that had been purified as CLPs and CMPs and the finding that the dendritic cell profiles were identical. The investigators concluded that the program for dendritic cells can operate independently of the myeloid and lymphoid pathways [52]. Many investigators have brought to attention trans-differentiation of one cell type into another, but this has not been demonstrated convincingly at a clonal level for marked hematopoietic cells.

Like HSCs, HPC decision-making in canonical models is a binary process. There is strong support of the view that cellular controls are bi-stable with cells switching from one steady state to an alternative steady state as driven by an external event or a change to an internal process. Binary tree-like maps invariably describe the development of entire organisms, for example, the newly hatched roundworm Caenorhabditis elegans [53], and various tissues, for example, neuronal-crest-derived cells [54]. A recent and complex gene regulatory/neural network-based analysis arrived at a binary-fork transition map for Caenorhabditis elegans and hematopoiesis, with decisions being driven by a combination of signals [55].

However, might HSCs be multi-stable and be able to process more than two exclusive options? Multi-stability exists for positive feedback systems and signaling pathways [56,57] and metabolic networks [58]. The transcription factors GATA1, GATA2, and PU.1 play key roles in the development of HSCs and HPCs. One option versus another is easier to model, and investigators first arrived at bi-stable models for a framework for the actions of the transcription factors. Bi-stable models were then embedded to achieve a tri-stable model, which was further modeled to encompass four mutually exclusive stable states. The findings from their modeling fitted experimental data [59]. We cannot, therefore, exclude that HSCs can process complex information regarding how they make a choice of lineage.

### 5.2. HPC Progression Is Gradual and Versatile

**Figure 3:**
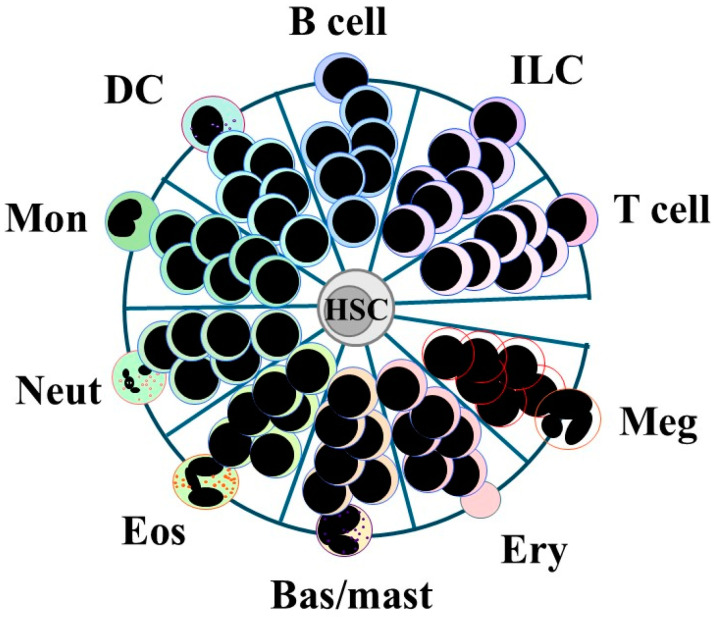
A continuum model for hematopoiesis. Lineage-affiliation is initiated as early as within HSCs whereby each either self-renews or makes a choice from a continuum of all end cell options that are directly available. An HSC then differentiates in a continuous manner. There are close relationships between developmental pathways, and HSCs having ‘chosen’ an affiliation can still change their mind to step sideways to an adjacent pathway. Meg, megakaryocytes; Ery, erythrocytes; Bas/mast, basophils/mast cells; Eos, eosinophils; Neut, neutrophils; Mon, monocytes; DC, dendritic cells; ILC, innate lymphoid cells.

The continuum model in Figure 3 shows that all end options are available directly to HSCs and that development to an end cell type is then a gradual and continuous process. There are no arrows to show a preferred route because a series of HPCs with different set potentials does not underlie progression. Having selected a cell lineage, HSCs and their offspring can still step sideways to an alternative, albeit closely related fate. In other words, developing cells are versatile and tend to switch most readily to a path that leads to their pairwise neighbor fate, with alternative fates having remained latent or clandestine. Support to versatility has been provided from RNA sequencing of more than 1600 mouse cells to examine the trajectories of HSCs and HPCs as these cells developed along the erythroid, granulocyte/macrophage, and lymphoid pathways. The construction of a single-cell resolution map for differentiation led investigators to conclude that trajectories are broad, which would allow cells to sidestep to the left or right of a chosen pathway [60].

In the continuum model, there are close relationships between the pathways for each cell lineage. They are like those shown in canonical models, which is not too surprising because placing neighbors in both models took into consideration the cohorts of options that were revealed by CFU cells and bi-potent normal and cell line cells [61]. Investigators arrived at a radius plot identical to that shown in Figure 3 from mathematical modeling of the networks that control the dynamics of blood formation and state transitions [62]. The placement of cell lineages in Figure 3 also took into consideration the shared usage of transcription factors [39]. Microarray profiling of gene expression, including the expression of transcription factors, within HPCs and mature cell types arrived at a similar pattern of near neighbors. They were erythrocytes, megakaryocytes, granulocytes/monocytes, dendritic cells, B cells, natural killer cells, and T cells [63].

Is the progression of HSCs towards uni-potent HPCs a continuous process? Investigators captured the transcriptional landscapes of multipotent (LSK) and oligopotent (Lin^−^, Sca−, Kit^+^) cells as they developed and the relationships between pathways by clonal barcoding of cells. These cells gave rise to nine cell types, namely megakaryocytes, erythrocytes, basophils, mast cells, eosinophils, neutrophils, monocytes, dendritic cells, and lymphoid precursors when cultured in conditions for cell growth and muti-lineage differentiation. Single-cell RNA sequencing was undertaken for cells that were sampled immediately and later. The transcriptional states of the least differentiated HPCs did not match a discrete hierarchy of oligopotent and intermediate HPCs and instead supported the view that developing cells lie along a continuum of states. Some clones exhibited uni-lineage differentiation and others multi-lineage differentiation. The transcriptional landscapes obtained for the cells that had appeared by 6 days in culture were used to construct a map for how multipotent HPCs had veered towards pathways. The near-neighbor pathways observed were for megakaryocyte, erythrocyte, mast cell, basophil, eosinophil, neutrophil, monocyte, migratory dendritic cell, plasmacytoid dendritic cell, and lymphoid development, like those shown in Figure 3. A continuum landscape was also seen for cells that had developed in vivo. This was revealed by barcoding LT-HSCs/ST-HSCs (Lin^−^, Sca^high^, kit^+^), culturing for two days, and then transplanting into irradiated mice. The near-neighbor pathways observed were for development towards erythrocytes, basophils, neutrophils, monocytes, dendritic cells, B cells, and T cells [64]. From findings for the lineage contribution of mouse HSCs and HPCs from transplantation studies, investigators concluded that native hematopoiesis is not entirely established by hierarchical HPC differentiation. The model they proposed has an initial broad landscape with pathways eventually emerging for megakaryocyte, erythrocyte, myeloid/granulocyte, myeloid/monocyte, and lymphocyte development [65].

Findings for human HPCs support the view that their development is a continuous process. This was revealed by constructing the developmental trajectories for the immediate progeny of HSCs (Lin^−^, CD34^+^, CD38^−^) and more differentiated HPCs (Lin^−^, CD34^+^, CD38^+^). Findings from single-cell RNA sequencing were combined with findings for single-cell culture differentiation outcomes. For the immediate progeny of HSCs (Lin^−^, CD34^+^, CD38^−^), there was an absence of clusters, and instead, these cells were a continuously connected entity. Clusters that conformed to distinct HPCs for each of the major hematopoietic cell types were observed for the more mature Lin^−^, CD34^+^, and CD38^+^ cells. From these findings, the investigators concluded that early hematopoiesis is best represented by a cellular continuum of low-primed undifferentiated (CLOUD)-HSCs/HPCs. They proposed that these cells gradually acquire lineage priming in multiple directions. A graphical summary of the findings showed that the erythroid, megakaryocyte, eosinophil/basophil/mast cell, neutrophil, monocyte/dendritic cell, and B cell pathways were near neighbors [66].

In 1957, Waddington proposed a metaphorical and epigenetic landscape for the development of primitive embryonic cells. He proposed that these cells develop from a broad upland, like continuum models. Developing cells then coursed downwards through bifurcating valleys with the hills to the valleys offering little chance of sideways escape to emerge as an alternative differentiated cell [67]. In this case, the likelihood of lateral transition by HPCs and reprogramming may become more restricted as development proceeds by virtue of end-cell-type programs becoming increasingly stable. HSCs and/or MPPs might, therefore, be more likely to be instructed to veer toward an alternative option(s) during their early development. The ligand to FLt3 has been proposed to have an instructive role at an early stage of HSC development. Instruction was dependent on ligand concentration because if the signal strength exceeded a certain threshold, level cells veered toward lymphoid and myeloid fates at the expense of megakaryocyte and erythroid development [68,69]. When FLt3 was overexpressed in megakaryocyte/erythroid HPCs, they veered toward granulocytes and macrophages [70]. A recent study has described the cellularity of human bone marrow relating to Flt3 ligand deficiency and concurred that if early HPCs do not receive sufficient Flt3 ligand stimulation they preferentially differentiate toward megakaryocyte and erythrocytes. The investigators concluded that the Flt3 ligand governs the development of partially overlapping lineages [71].

## 6. Can Findings Be Reconciled to a Consensus Model?

A view for many years has been that the only long-term self-renewing cells in the hematopoietic system are HSCs. The term ‘immortality’ has been too loosely applied to these cells because this has not been tested in a strict sense. Now there is also good evidence to support extending the capacity for self-maintenance to multi-lineage biased and even lineage affiliated cells whereby these cells can make a substantial contribution to hematopoiesis to replenish an end cell type(s). They may do so to a greater extent during emergency hematopoiesis as opposed to steady state. Vector integration sites were used as markers of clonal identity in studies that used lentivirus HSCs for gene therapy of metachromatic leukodystrophy, Wiskott–Aldrich syndrome, and β-thalassemia. For all conditions, 50% of the transplanted clones showed a multi-lineage output. The remaining clones showed a preferential lineage output, which was myeloid for metachromatic leukodystrophy, lymphoid for Wiskott–Aldrich syndrome, and erythroid for β-thalassemia. From these findings, lineage-committed HSCs/HPCs, including uni-lineage cells, had persisted for several years post-transplantation [72]. Again, the capacity for self-renewal does not draw a clear line between cells that have been ring-fenced for many years as HSCs versus HPCs.

In early studies, the lineage potentials of HPCs were determined from the diverse combinations of mature cells that were seen when bone marrow cells were dispersed in semi-solid medium to give rise to CFUs and transferred to an irradiated mice to give rise to spleen CFUs. Findings from these studies provided the backbone for arriving at canonical models. The placement of lineage pathways close to one another is quite similar in canonical models and the continuum model. Therefore, there are clearly close relationships between cell lineages. Various oligopotent progenitors can be reconciled with a continuum model because HSCs/HPCs developmental progression is a versatile process. They were mapped to the continuum model by assuming that the different cell types generated by single cells in the CFU assay relate to limited transitions to another pathway as colony cell numbers increase [73].

More recent studies of the lineage potentials of HSCs and HPCs have examined the molecular feature of single HPCs/HSCs globally, made use of clonal bar-coding to track their development, and sub-fractionated HSCs for transplantation. Findings have led to a much more complex topology and to the developmental progression of HSCs/HPCs. Features of continuum models are that multiple pathways arise directly from HSCs, the developmental progression of cells is a continuous process, and cells that have chosen an option can still adopt another pathway. This versatility questions whether irreversible states of commitment exist. It has been known for many years that cells that are well on their way to becoming T cells can generate other cell types. Early T cell progenitor (double negative (DN)) cells express neither CD4 nor CD8. Kit^hi^ DN1 and DN2 cells can give rise to natural killer and myeloid cells [74,75,76]. Mature cells can change their functional characteristics as seen in the interconversion of CD4+ T effector cells. T helper 2 cells can give rise to follicular T helper cells [77] and regulatory T cells can convert to pro-inflammatory T helper 17 cells [78].

## 7. Concluding Remarks

Some 75 years have elapsed since the description of HSCs. Presently, we might conclude that drawing a hierarchical model for a series of hierarchical progenitors that have strict lineage options is an oversimplification of the complex ability and adaptability of hematopoiesis. We might expect versatility/flexibility because hematopoiesis must meet the demands of both steady state and emergency circumstances. However, we are still learning how to combine aspects of various models to arrive at a consensus as to the process of hematopoiesis. This is important to what the difference is between the behavior of normal and leukemia stem cells. Leukemia stem cells are rare cells that sustain leukemia, and like HSCs, they generate a hierarchy of cells. The different acute leukemias most likely arise from the transformation of an HSC. The self-renewal ability of HSCs places them most at risk of transformation regarding the generation of a cell that can sustain leukemia. The leukemias are categorized according to the cellular types that are present, namely myeloblastic, granulocytic, erythroblastic, monocytic, lymphoblastic, etc. The existence of lineage-affiliated HSCs may explain why a transformed HSC is restricted to generating cells of just one cell type. An alternative view relates to each of the leukemias having a signature oncogene, and there is good evidence to support the view that the oncogene instructs cell lineage [79,80,81,82]. Hence, a resolve to the precise nature of hematopoiesis is inextricably tied to efforts to unravel the nature of leukemia.
